# Interaction mechanism of aloe-emodin with trypsin: molecular structure–affinity relationship and effect on biological activities

**DOI:** 10.1039/d0ra02712j

**Published:** 2020-06-02

**Authors:** Guoyan Ren, He Sun, Gen Li, Jinling Fan, Lin Du, Guoting Cui

**Affiliations:** College of Food and Bioengineering, Henan University of Science and Technology Luoyang 471023 China renguoyan@163.com +86-15937969597; Henan Engineering Research Center of Food Material Luoyang 471023 China; National Demonstration Center for Experimental Food Processing and Safety Education Luoyang 471023 China

## Abstract

The molecular mechanism of interaction between aloe-emodin (AE) and trypsin was investigated, exhibiting remarkable outcomes. To detect the interaction mechanism, the binding of AE with trypsin was examined by a multi-spectroscopy and molecular docking method. Results showed that the binding of AE and trypsin would lead to static quenching and their binding forces were van der Waals forces and hydrogen bonding. The results of simultaneous and three-dimensional fluorescence spectroscopy showed that the combination of AE and trypsin caused changes in the microenvironment around the trypsin fluorophore, which might change the spatial structure of trypsin. FT-IR spectroscopy showed that the contents of α-helix and β-turn in trypsin were decreased and the contents of β-sheet, random coil and antiparallel β-sheet were increased. Moreover, all these experimental results were verified and reasonably explained by molecular docking results. We also investigated the enzyme activity of trypsin and the antioxidant activity of AE. The results showed that both the enzyme activity of trypsin and the antioxidant activity of AE were decreased after interaction between AE and trypsin. The findings outlined in this study should elucidate the molecular mechanisms of interaction between AE and trypsin and contribute to making full use of AE in the food industry.

## Abstract 2

The mechanism of interaction between AE and trypsin was studied firstly. The biological activity of both decreased after the interaction. These results provide a basis for the development and utilization of AE.

## Graphical abstract

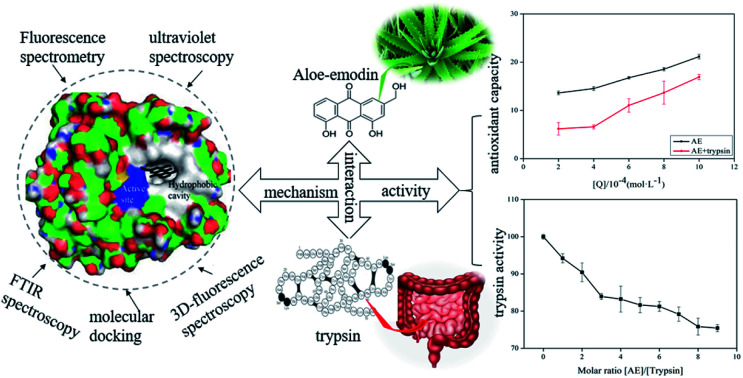

## 1. Introduction

In recent years, the interaction between small molecules and proteases has become a focus of international attention, which is closely linked to the in-depth understanding of medicine, chemistry, food science, toxicology and biology.^1,2^ Trypsin (EC 3.4.21.4, 23.3 kDa, containing 223 amino acids, including 4 tryptophan residues, 10 tyrosine residues, and 6 phenylalanine residues) is a typical serine protease.^3^ Trypsin is an important protease in the human digestive system. It has a variety of biological functions in the organisms, including digestion and deconstruction of food proteins, and participation in physiological processes such as apoptosis, hemostasis, signal transduction, reproduction and immune response (Zhang, Zhou, Cao, & Wang, 2013).^4^ The biological function of trypsin was closely connected with its catalytic activity. Numerous studies showed that small molecules extracted from fruits, healthy foods and energy drinks, such as methotrexate,^5^ caffeine, theophylline and resveratrol,^6,7^ interact with trypsin to affect its conformation and thus its catalytic activity, leading to changes in its biological function. However, up to now, there has failed to report on the interaction between AE and trypsin.

**Fig. 1:**
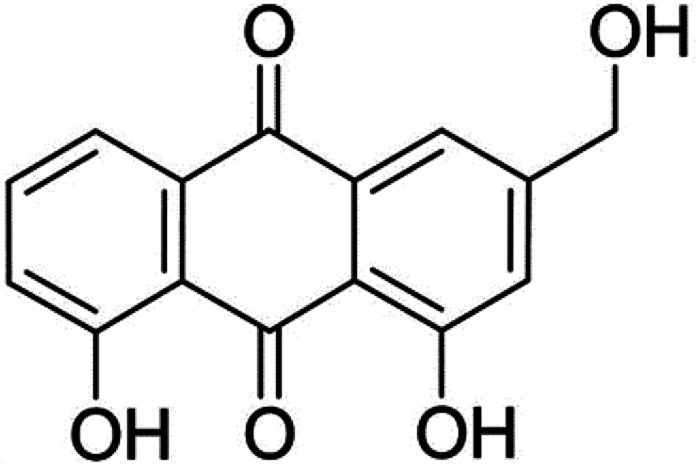
Structure of aloe-emodin.

Aloe-emodin (AE, Fig. 1) was a natural anthraquinone compound containing multiple phenolic hydroxyl groups, which had anti-cancer, liver protection, anti-oxidation, anti-fungal, laxative and other pharmacological activities, and was widely used in food, biology, medicine, chemical industry and other fields.^8–11^ AE was currently used by the food industry as a functional food additive in wine, beverages, flour products and catering industries. After entering the human body with food, AE would interact with trypsin, which might affect the spatial structure and biological activity of AE and trypsin, leading to changes in their biological functions. In order to utilize the bioactivity of AE and trypsin effectively, it was necessary to elucidate the mechanism of AE–trypsin interaction.

In this paper, a variety of spectroscopy and molecular docking techniques were used to analyze the specific action process of AE and trypsin, as well as the effects on enzyme structure, activity and AE antioxidant activity. This would provide an important theoretical basis for revealing the interaction mechanism of AE and trypsin and its effect on biological activity.

## 2. Materials and methods

### 2.1 Chemicals

Bovine trypsin (T105531) was purchased from Aladdin Reagent Co., Ltd. (Shanghai, China) and used directly in the experiment. AE was purchased from Sangon Biotech Co., Ltd. (Shanghai, China). Tris–HCL buffer (AR grade, pH 7.4, 0.05 mol L^−1^) was obtained from Shanghai Qiangshun Chemical Reagent Co., Ltd (Shanghai, China). Stock solution of trypsin (2.5 × 10^−4^ mol L^−1^) was prepared in the Tris–HCL buffer. Stock solution of AE (1.0 × 10^−4^ mol L^−1^) was prepared in low concentration methanol (<5%, v/v). All solution was stored at 4 °C in a refrigerator. The deionized water applied in the experiment was filtered through a purification system (USA Milli-Q). Other reagents were analytical grade.

### 2.2 UV-visible (UV-Vis) absorption spectroscopy

UV-Vis absorption spectroscopy were studied by 2600 UV-visible Spectrophotometer (Shimadzu Corporation, Japan) with 1.0 cm quartz cell and recorded in the 185–700 nm range. Spectral analysis was performed using software in the instrument. Prepared AE–trypsin complex (molar ratio 1 : 1), mixed well, and detected after 30 minutes incubation at room temperature. Ultraviolet absorption spectra of trypsin solution (1.0 × 10^−5^ mol L^−1^), AE–trypsin complex solution and AE solution (1.0 × 10^−5^ mol L^−1^) were measured respectively. Ultrapure water was used as blank control.

### 2.3 Fluorescence spectroscopic measurements

A Cary Eclipse Fluorescence Spectrophotometer (Agilent, USA) with a 1.0 cm four-sided transparent quartz test cuvette was used for steady-state fluorescence studies. Tris–HCl buffer was used to dilute the trypsin concentration to 2.5 × 10^−5^ mol L^−1^. Prepared mixtures of AE and trypsin with their molar ratios being 0, 0.25, 0.5, 0.75, 1.25, 1.5, 1.75, 2 and 2.25, respectively. All solutions were mixed uniformly and detected after 10 minutes of storage at room temperature. At three different temperatures (298, 304 and 310 K), the emission fluorescence curves of trypsin with and without AE were recorded in the wavelength range of 300–500 nm. The excitation wavelength was 280 nm, and the excitation and emission slit widths were set to 5 nm. The scanning speed was 600 nm min^−1^. In this study, all fluorescence intensities were corrected according to the following formula:^12^

1 *F*_corr_ = *F*_obs_ × 10^(OD_ex_+OD_em_)/2^

*F*_corr_ was the corrected fluorescence intensity, as observed by *F*_obs_. OD_ex_ was the system fluorescence absorption intensity at the excitation wavelength, and OD_em_ was the system fluorescence absorption intensity at the emission wavelength.

### 2.4 Synchronous fluorescence spectroscopy

The synchronous fluorescence measurements were carried out in the same instrument (a Cary Eclipse Fluorescence Spectrometer). The fluorescence emission curves of trypsin fluorescence groups in the presence and absence of AE were detected at 298 K. The fixed excitation wavelength and emission wavelength spacing were Δ*λ* = 15 nm (for Tyr) and Δ*λ* = 60 nm (for Try), respectively, and the excitation wavelength range was 200–500 nm. Other experimental parameters were the same as in the steady state fluorescence studies.

### 2.5 Three-dimensional (3D) fluorescence spectroscopy

The 3D fluorescence spectroscopy were performed on the same instrument (Agilent Cary Eclipse Fluorescence Spectrophotometer). The 3D fluorescence spectroscopy (298 K) of trypsin and AE–trypsin (1 : 1 molar ratio) were detected in the range of 200–400 nm excitation wavelength. The emission spectrum was monitored in 200–500 nm. Other experimental parameters were the same as those of steady-state fluorescence experiments.

### 2.6 FT-IR spectroscopy

The FT-IR spectroscopy was performed according to previous studies.^13^ The Vertex 70 FT-IR spectrometer (Bruker, Germany) was used to measure the FT-IR spectroscopy of trypsin and AE–trypsin complex. Used OMNIC 8.2 data processing software. At room temperature and an open state, automatic vapor correction was performed to collect background data.

### 2.7 Molecular docking

Crystal structure of bovine trypsin was from protein database (PDB Entry: 2ZQ1), and crystal structure of AE ligand was from PubChem compound database (PubChem CID: 10207). AE as a flexible molecule docked with trypsin (rigid molecule). Before docking, used DS visualizer (v17.2.0.16349) to remove water, add hydrogen atoms, and repair all amino acid residues. The interaction between trypsin and AE was studied by Autodock 4.2.6 program. Several important parameters were set as follows: the Gird was 120 × 126 × 126 Å, and the grid spacing was 0.375 Å. The running time was 1000, the default maximum generation was 27 000 was 2.5 million, and the energy evaluation. The Lamarck Genetic Algorithm was applied to select the possible ligand–protein conformation. PyMol (version 1.5.0.3) was utilized to display the docking results.

### 2.8 Trypsin catalytic activity assay

The detection method of trypsin catalytic activity with and without AE referred to our description in previous research.^7,13^ The molar ratio of AE to trypsin was 0, 0.25, 0.5, 0.75, 1.25, 1.5, 1.75, 2 and 2.25. Each experiment was performed in triplicate. The catalytic activity of trypsin could be calculated according to formula (2):

2 Relavtive activity (%) = (*a*/*A*) × 100%

where *A* was the enzyme activity of trypsin without AE and a was the enzyme activity of trypsin with AE.

### 2.9 Antioxidant activity assay

The detection methods of antioxidant activity of AE with and without trypsin were according to the methods described in our previous published papers.^7,13^ The scavenging rate of superoxide anion radical and DPPH (1,1-diphenyl-2-picrylhydrazine) were used to evaluate the antioxidant activity of AE.

## 3. Results and discussions

### 3.1 UV-visible absorption spectroscopy investigations

**Fig. 2:**
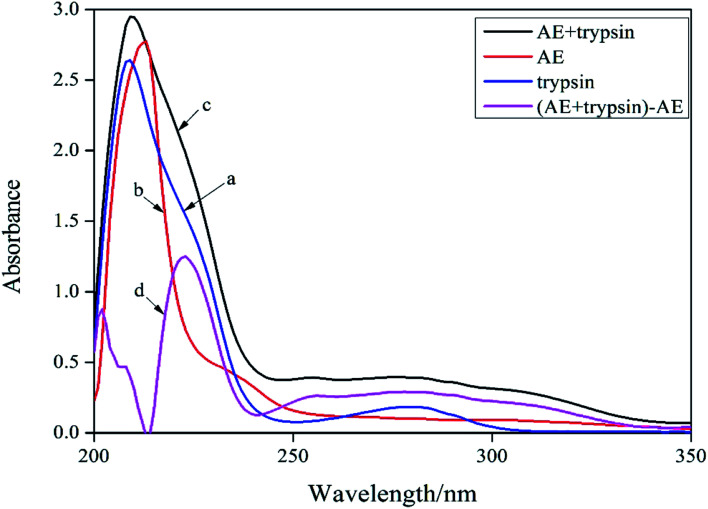
UV-vis absorption spectra of trypsin and AE–trypsin. *C*_(trypsin)_ = 2.5 × 10^−5^ mol L^−1^, *C*_(AE)_ = 3.0 × 10^−5^ mol L^−1^, *T* = 298 K.

UV-vis absorption spectroscopy was a common method for studying the structure change and formation of complexes because of its simplicity and effectiveness.^14^ Changes of the microenvironment around trypsin chromophore could lead to changes in protein absorption spectrum. Therefore, the UV-visible absorption spectrum of protein could be used to study the changes of protein structure in solution. In the ultraviolet absorption spectrum of proteins, the weak absorption peak at 280 nm caused by aromatic amino acids was usually employed to reflect conformation changes of protein. The absorption spectra of trypsin (curve *a*) and [AE + trypsin] − AE (curve *d*) were shown in Fig. 2. The curve *a* and curve *d* should be the same if AE did not interact with trypsin. By comparing the curve *a* and the curve *d* in Fig. 2, it displayed the absorption peak of trypsin at 280 nm became wider, the peak height increased slightly, and appeared a red shift. This result suggested that there was an interaction between AE and trypsin and the formation of new complexes, which led to a change in the trypsin conformation. However, the combination mode needed to be further confirmed.

### 3.2 Fluorescence quenching of trypsin by AE

**Fig. 3:**
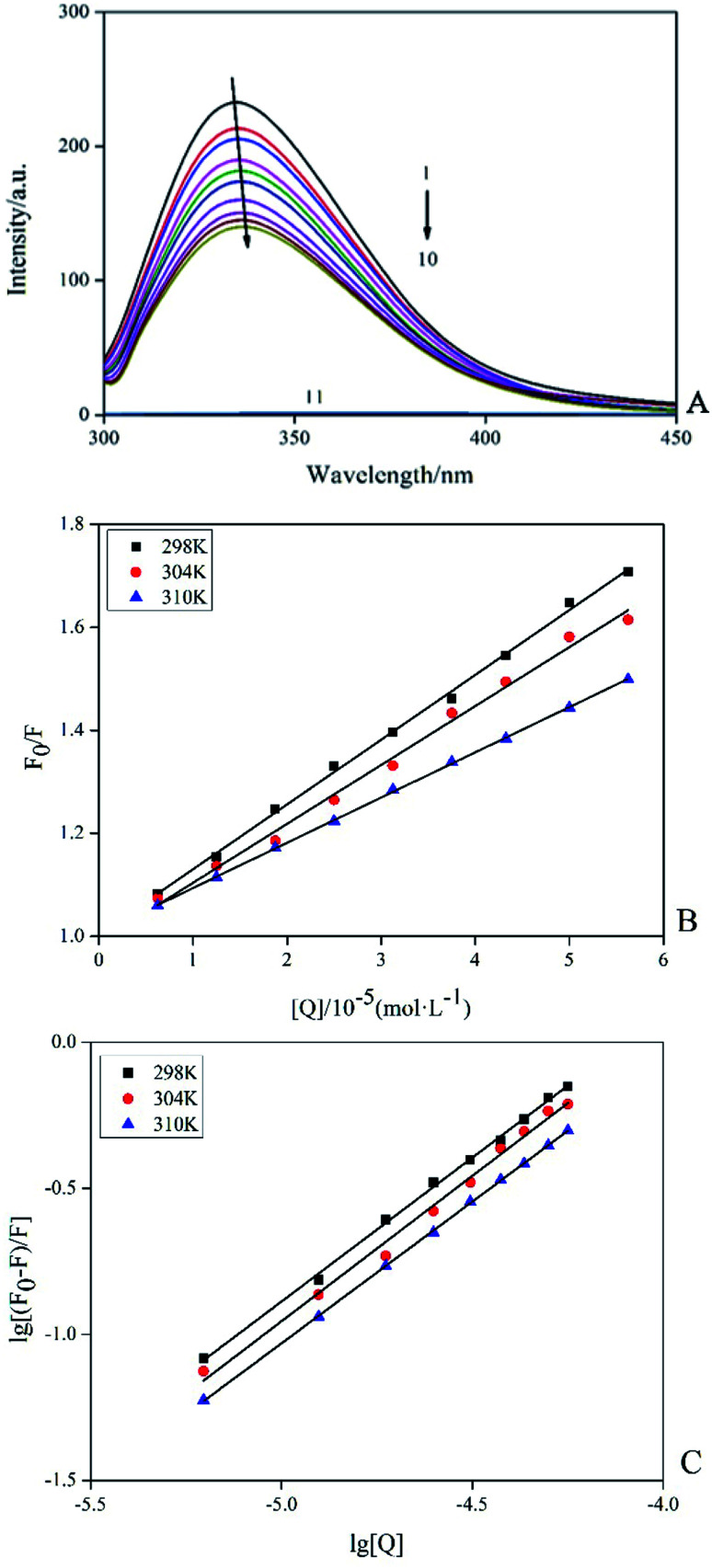
Fluorescence spectra of trypsin (2.5 × 10^−5^ mol L^−1^) with and without AE (1 → 10: 0, 0.25, 0.5, 0.75, 1.25, 1.5, 1.75, 2 and 2.25, AE/trypsin, molar ratio; 11 : AE) (A). The Stern–Volmer plots of trypsin with the addition AE at various temperatures (B). Double logarithm regression plots of AE–trypsin at various temperatures (C).

Among the methods for studying the interaction between proteins and ligands, fluorescence detection had become an effective method because of its high sensitivity, rapidity and ease of implementation. This method could provide characteristic information of binding ligand to protein, such as binding mechanism, binding pattern, binding site, binding constant and intermolecular force.^15^ Fluorescence quenching was the process of adding a quencher to reduce the fluorescence intensity of the substance. The inherent fluorescence of trypsin was mainly derived from 4 Trp and 10 Tyr residues and the fluorescence pattern changes of these fluorophores could provide information for the conformational changes of induced trypsin by AE. Fig. 3A showed the fluorescence emission spectra (298 K) of trypsin in the presence and absence of different concentrations of AE. When excited at 285 nm, trypsin had a strong fluorescence emission peak at 342 nm. The fluorescence intensity of trypsin decreased with the increase of AE concentration. The maximum emission wavelength appeared a red shift. This suggested that the microenvironment around Trp and Tyr residues of trypsin changed after the interaction between AE and trypsin, leading to the fluorescence quenching of trypsin.^16^

According to different quenching mechanisms, fluorescence quenching modes were divided into dynamic quenching, static quenching, and dynamic and static mixed quenching.^17^ The quenching mechanism of AE–trypsin binding could be determined by analyzing the fluorescence data using the Stern–Volmer equation:

3 *F*_0_/*F* = 1 + *K*_q_*τ*_0_[*Q*] = 1 + *K*_sv_[*Q*]

Fig. 3B shows the Stern–Volmer diagram of the quenching effect of AE on trypsin fluorescence. The results showed that this method had a good linearity curve, which revealed that AE and trypsin interaction was a single type of quenching.^18^ All data were listed in Table 1. The negative correlation between *K*_sv_ (the quench constant of fluorescence between AE and trypsin) value and temperature indicated AE quenched trypsin fluorescence through a static quenching mechanism possibly. All *K*_q_ (the quenching rate constant or bimolecular quenching constant) values were greater than the maximum molecular collision coefficient (2.0 × 10^10^ M^−1^ s^−1^), offering additional evidence for static quenching mechanism.^19^

**Table 1:** Quenching rate constants and correlation coefficients of AE–trypsin

*T*/*K*	*K*_sv_ (L mol^−1^ S^−1^)	*K*_q_ (L mol^−1^ S^−1^)	*R* ^2^	*K* (L mol^−1^)	*n*	*R*	Δ*G* (kJ mol^−1^)	Δ*H* (kJ mol^−1^)	Δ*S* (J mol^−1^·K^−1^)
298	1.2582 × 10^4^	1.2582 × 10^12^	0.9979	1.0898 × 10^4^	0.9849	0.9983	−23.0324		
304	1.1441 × 10^4^	1.1441 × 10^12^	0.9926	1.0507 × 10^4^	0.9953	0.9921	−23.4037	−32.076	−29.749
310	8.7822 × 10^3^	8.7822 × 10^11^	0.9998	6.5832 × 10^3^	0.9698	0.9998	−22.6607		

For static quenching interactions, the binding constant between AE and trypsin (*K*) and binding sites (*n*) between AE and trypsin could be calculated from the double logarithmic equation:^20^

4 lg[(*F*_0_ − *F*)/*F*] = lg *K* + *n* lg[*Q*]

where [*Q*] was the quencher concentration, *K* was the binding constant between AE and trypsin constant, *n* was the number of binding sites. The *K* values (about 10^4^, Table 1) at different temperatures were less than the strong affinity coefficient (10 ^6^ to 10^8^ M^−1^) and decreased with the increasing temperature (Fig. 3C), indicated the formation of a less stability complex between trypsin and AE. Values of *n* close to 1 implied that AE had only one binding site to the trypsin.^21^

### 3.3 Calculation of thermodynamic parameters

The interaction forces between biological molecules and small molecules included hydrogen bonding, electrostatic interaction, van der Waals interaction, hydrophobicity and so on. Thermodynamic parameters were the main basis to prove the bonding force. Values of enthalpy change Δ*H*, and entropy change Δ*S* were calculated with van't Hoff eqn (5) and values of Gibbs free energy change Δ*G* was calculated by eqn (6).^22^

5 ln *K* = −Δ*H*/*RT* + Δ*S*/*R*

6 Δ*G* = −*RT* ln *K* = Δ*H* − *T*Δ*S*

where *R* was the gas constant, *T* was the experimental temperature, *K* was the association constant. Δ*H* and Δ*S* can be calculated according to eqn (5).

The Δ*G* < 0 (in Table 1) implied that the process of AE binding to trypsin was spontaneous exothermic.^23^ During the interaction between AE and trypsin, the values of Δ*H* (−32.067 kJ mol^−1^) and Δ*S* (−29.749 J mol^−1^ K^−1^) both were negative, suggested that van der Waals force and hydrogen bonding were the main interaction force of AE–trypsin complex in the light of the theory of Ross and Olsson.^24^

### 3.4 Synchronous fluorescence spectra

**Fig. 4:**
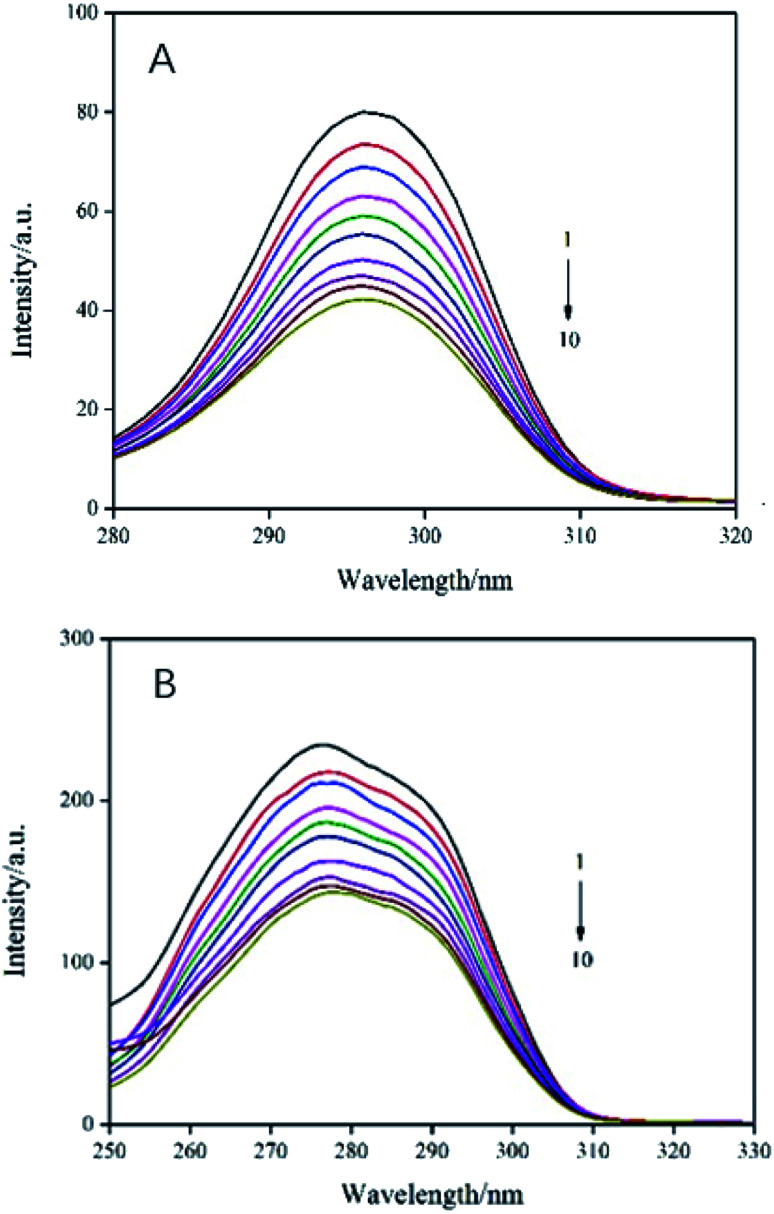
Synchronous fluorescence spectra of AE–trypsin. (A) Δ*λ* = 15 nm and (B) Δ*λ* = 60 nm, *T* = 298 K. *C*_(trypsin)_ = 2.5 × 10^−5^ mol L^−1^, AE/trypsin at molar ratio (0, 0.25, 0.5, 0.75, 1.25, 1.5, 1.75, 2 and 2.25).

In order to evaluate the molecular microenvironment near the functional fluorophore (Trp and Tyr residues) of trypsin, synchronous scanning spectroscopy studies were performed by simultaneously scanning excitation and emission monochromators at constant wavelength differences Δ*λ* = 60 nm for Trp residue and Δ*λ* = 15 nm for Tyr residue. The synchronous fluorescence spectra of trypsin and AE was showed in Fig. 4. The interaction between the emission peak of trypsin and AE showed a red shift of 1 nm (from 277 nm to 278 nm), which indicated that hydrophobicity decreased and polarity increased in the Trp microenvironment due to AE insertion.^25^ In addition, with the increase of AE concentration, the fluorescence intensity at Δ*λ* = 60 nm decreased steadily, which supported the fluorescence quenching when AE interacts with trypsin. For Tyr residue with Δ*λ* = 15 nm, the emission peak decreased with the increase of AE concentration, but the maximum emission wavelength did not change significantly, indicating that the microenvironment around Tyr residue was less disturbed. Therefore, it could be said that the possible binding site of AE was closer to Trp residue than to Tyr residue in trypsin.^26^

### 3.5 The 3D fluorescence spectroscopy

**Fig. 5:**
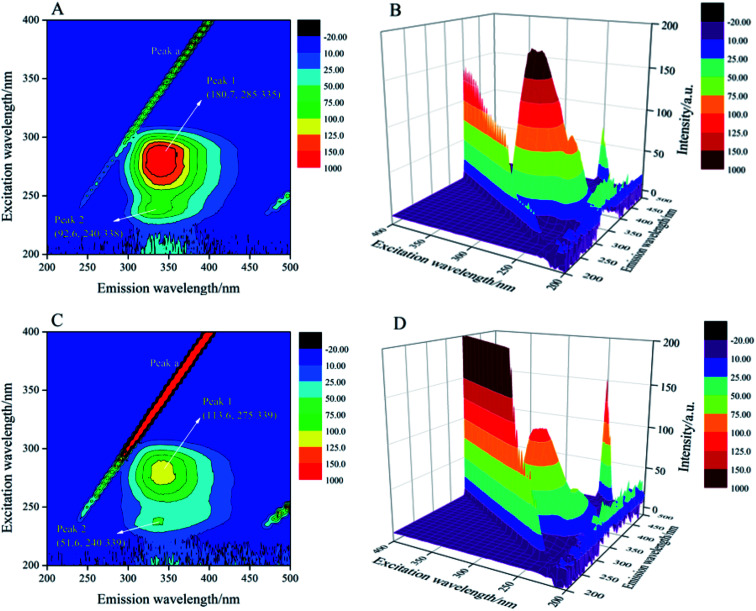
The 3D fluorescence spectra of trypsin with and without AE (A–D). Trypsin/AE = 1 : 1 *C* = 2.5 × 10^−5^ mol L^−1^, *T* = 298 K.

The 3D fluorescence spectroscopy was a more scientific and effective method to provide details of changes in protein microenvironment and spatial conformation. The 3D fluorescence spectra of trypsin and AE–trypsin complexes were showed in Fig. 5. The 3D fluorescence spectra of trypsin emerged four characteristic peaks, of which peak *a* (*λ*_ex_ = *λ*_em_) was the first-order Rayleigh scattering peak, and peak *b* (2*λ*_ex_ = *λ*_em_) was the second-order Rayleigh scattering peak. Both were system-generated peaks. Peak 1 (*λ*_ex_: 285 nm; *λ*_em_: 340 nm) was the intrinsic fluorescence spectral behavior of the aromatic amino acids (Trp and Tyr residues) in trypsin due to the π–π* transition of the electron. Peak 2 (*λ*_ex_: 275 nm; *λ*_em_: 339 nm) was the excitation of the highly excited electronic states of the aromatic residues of trypsin. For the reason that, when the excitation wavelength was 285 nm, the changes of peaks 1 and 2 that could reflect the local environmental changes of Trp and Tyr aromatic residues in trypsin should be consistent.^27^ It could be seen from Fig. 5A and C that when AE interacted with trypsin, the intensity of peak 1 was significantly reduced from 180.7 to 113.6, and accompanied by a red shift phenomenon from 335 to 339 nm. The intensity of peak 2 decreased significantly from 92.6 to 51.6, with a slight red shift from 338 nm to 339 nm. This indicated that Tyr and Trp residues had moved from a non-polar environment to a polar environment.^28^ These results were tallied with previous experimental results.

### 3.6 FT-IR spectroscopy

**Fig. 6:**
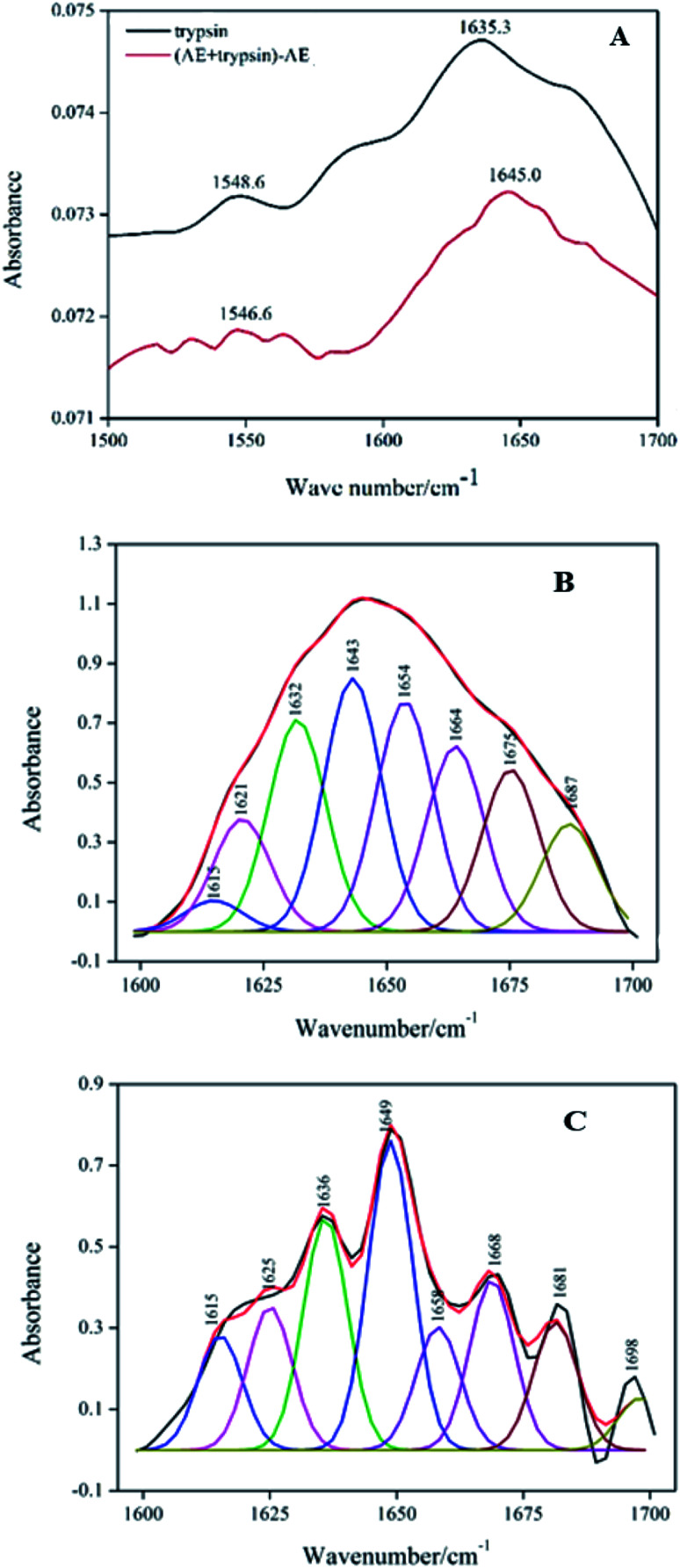
FT-IR spectra of trypsin with the absence and presence AE (A). Deconvolution of trypsin infrared spectrum (B). Deconvolution of (AE + trypsin) − AE infrared spectrum (C).

FT-IR spectroscopy was commonly used to evaluate changes in the secondary structural composition of proteins and ligand–protein complexes under various physical and chemical conditions.^29^Fig. 6A displayed the spectral characteristics in the amide I (1700–1600 cm^−1^) and amide II (1600–1500 cm^−1^) regions of trypsin and AE–trypsin (molar ratio 1 : 1) complexes. By comparing FT-IR spectra of trypsin to that of AE–trypsin, it was found that the peak position and peak shape of the amide I band and the amide II band changed, indicating that the binding of AE resulted in the change of the secondary structure of trypsin. Because of the higher conformation sensitivity of amide I region, quantitative analysis was conducted according to the procedure in the literature^30^ to calculate the composition of various secondary structures: antiparallel β-sheet (1680–1690 cm^−1^), β-turn (1660–1680 cm^−1^), α-helix (1650–1660 cm^−1^), random coils (1640–1650 cm^−1^) and β-sheets (1610–1640 cm^−1^).^31^ The amide I bands of trypsin (Fig. 6B) and AE–trypsin (Fig. 6C) were treated with self-convolution and second derivative, respectively, to obtain the composition of trypsin secondary structure (Table 2). As can be seen from Table 2, after AE binding, the α-helix and β-turn contents of trypsin decreased by 7.74% and 12.89%, respectively, while the β-sheet, random coil and antiparallel β-sheet contents increased by 12.73%, 5.73% and 2.17%, respectively. Such results implied the spatial structure of trypsin after AE binding became loose, and polar groups inside the trypsin molecule were exposed, leading to increased polarity and decreased hydrophobicity of trypsin. These results corroborated the previous fluorescence and UV-vis spectra experiments.

**Table 2:** The influence of AE on the secondary structure of trypsin

System	α-Helix (%)	Antiparalled β-sheet (%)	β-Sheet (%)	β-Turn (%)	Random coil (%)
Free trypsin	17.81	8.31	27.45	26.85	19.58
[AE + trypsin] − AE	10.07	10.48	40.18	13.96	25.31

### 3.7 Molecular docking

**Fig. 7:**
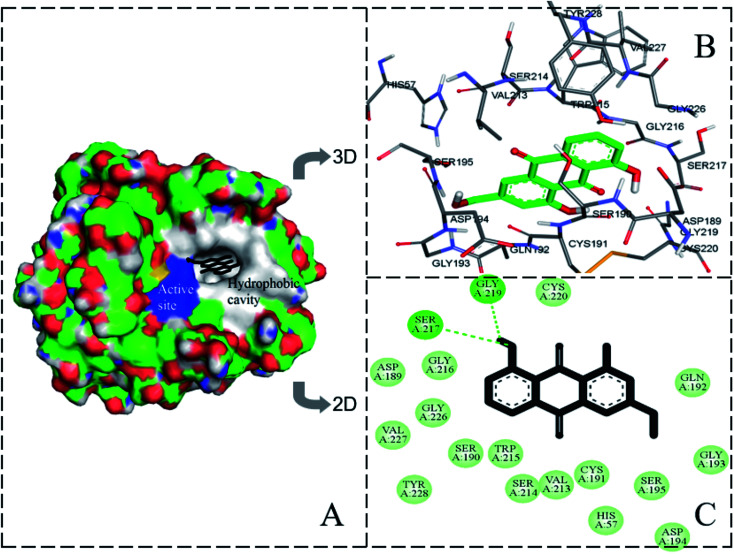
The molecular docking result of AE–trypsin complex. (A) Stereo view of the docking conformation of AE–trypsin complex. (B) Local amplification of the binding site and residues around AE. (C) The hydrogen bonds between AE and residues of trypsin.

Molecular docking further confirmed the experimental observations and established possible AE binding sites in the geometric structure of trypsin. The lowest binding energy and highest binding affinity conformation of AE–trypsin compounds was chosen to further analysis. The experimental free binding energy of −23.032 kJ mol^−1^ was close to the lowest binding energy of −26.375 kJ mol^−1^, suggested the reliability of molecular docking.^15^ As could be seen from Fig. 7, the site where AE binded to trypsin was located in the S1 pocket of hydrophobic groove. Trypsin residues around the AE molecule include His57, Asp189, Cys191, Ser190, Gly193, Gln192, Ser195, Asp194, Val213, Trp215, Ser214, Gly216, Cys220, Gly226, Ser217, Gly219, Val227 and Tyr228. There were two hydrogen bonds between Ser217 and Gly219 of trypsin and hydroxyl groups of AE (distances 4 Å involved). It could be seen that the main interaction forces in the binding process of AE to trypsin were hydrogen bonding and van der Waals force. This was coincided with the experimental results of previous thermodynamic analysis. It was also the intervention of non-covalent bonds destroyed the force of maintaining the original spatial conformation of trypsin, causing the spatial microstructure of trypsin to change.^32^ The position of AE was 3.5 Å and 3.9 Å from the Trp residue and the Tyr residue in trypsin, respectively, indicating that the position of AE interaction with trypsin was closer to the Trp residue. These conclusions agreed with experimental results of three-dimensional fluorescence, FT-IR and synchronous fluorescence spectroscopy. The molecular docking results were mutually verified with the previous spectroscopy experiment results, which could provide accurate information for the interaction mechanism between AE and trypsin.

### 3.8 Trypsin activity

**Fig. 8:**
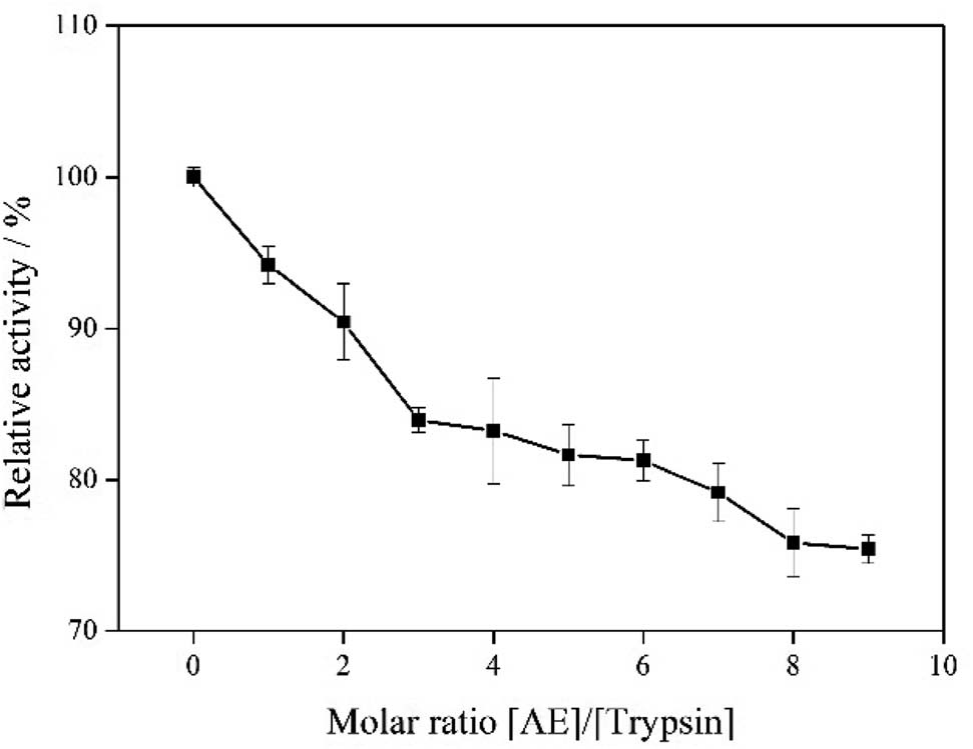
The catalytic activity of trypsin with addition of AE.

The effect of AE on trypsin activity was evaluated by measuring the catalytic activity of trypsin with and without AE *in vitro*. As shown in Fig. 8, the catalytic activity of trypsin continued to decrease as the concentration of AE increased, which indicated that AE had an inhibitory effect on the catalytic activity of trypsin. The catalytic mechanism of trypsin was very like that of other serine proteases. In the three-dimensional structure of bovine trypsin, it was composed of six disulfide bonds connected by two regions of similar size. Each domain consisted of six antiparallel β-sheets. Like other serine proteases, the catalytic activity of trypsin was influenced by two sites: one was the catalytic site composed of His57, Asp102 and ser195, the other was the main substrate binding site (S1 binding hydrophobic pocket) containing 189–195, 214–220 and 225–228 residues. The realization of trypsin activity was accomplished by these two parts.^33,34^ However, due to the different structure of ligand, the binding sites of ligand and trypsin were different, resulting in different effects on the catalytic activity of trypsin. Some ligands enhanced the catalytic activity of trypsin, while some ligands inhibited the catalytic activity of trypsin.^35,36^ In our previous molecular docking experiments on the interaction between resveratrol and trypsin, we found that resveratrol, as a ligand, binded in the hydrophobic pocket of trypsin, resulting in a decrease in the catalytic activity of trypsin.^7^ In this paper, the results of interaction between AE and trypsin showed that AE was also bound in the substrate binding pocket of trypsin and decreased the catalytic activity of trypsin, which was consistent with the previous studies. In addition, the interaction between AE and trypsin caused changes in the spatial structure of trypsin (Experimental results of spectroscopy), and might also lead to the decrease of trypsin catalytic activity.^37^

### 3.9 Antioxidant activities of AE

**Fig. 9:**
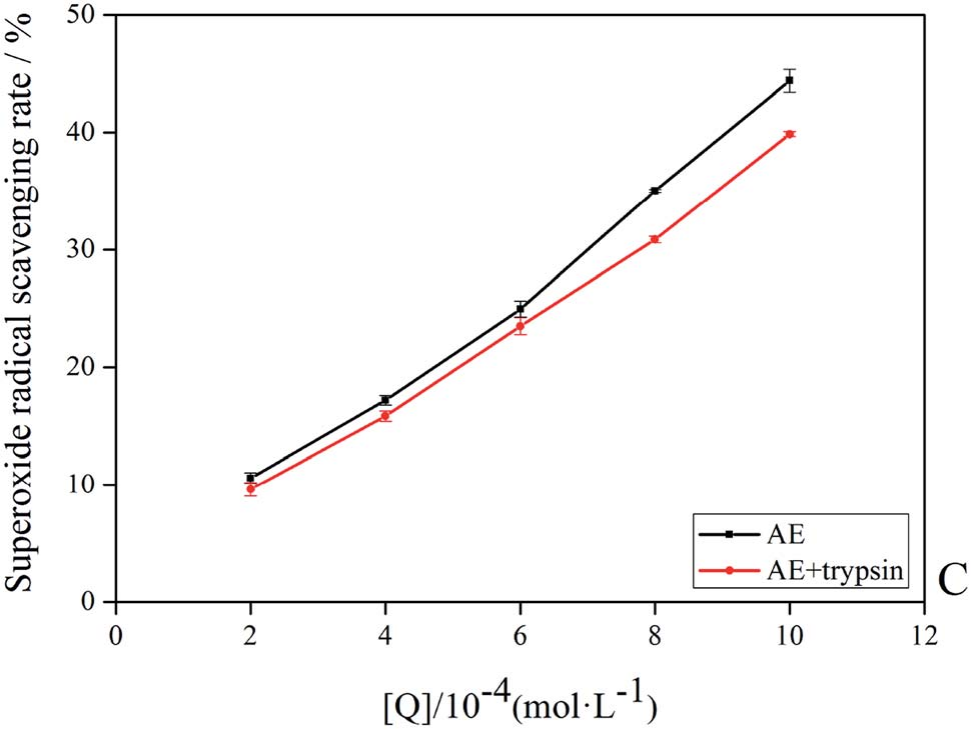
The superoxide anion radicals scavenging rate of AE and AE–trypsin complex.

**Fig. 10:**
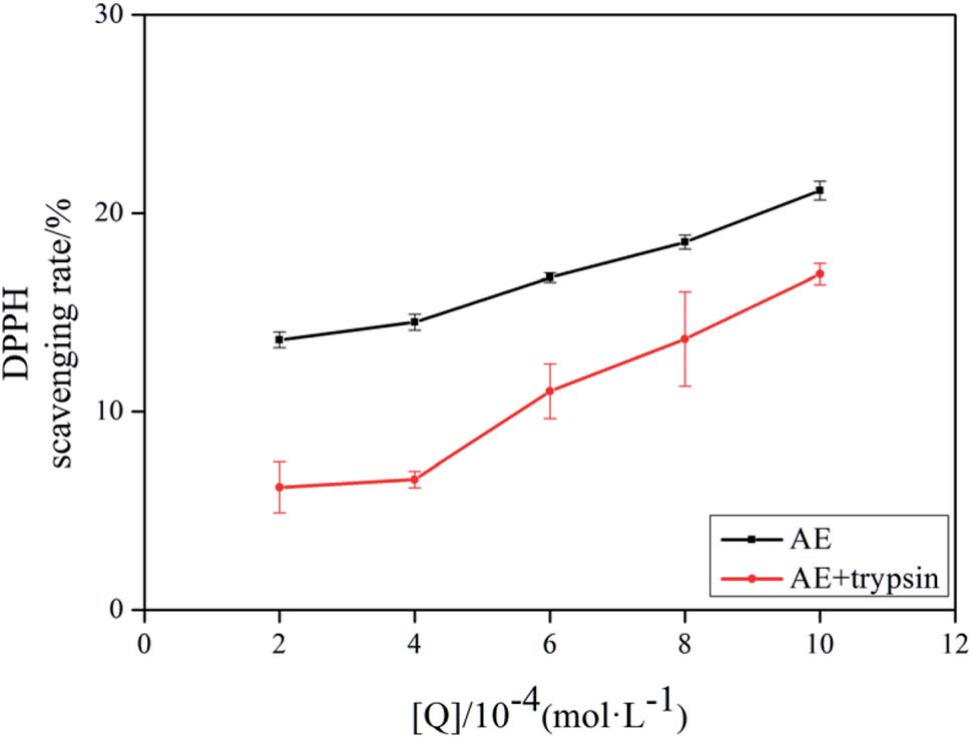
The DPPH scavenging rate of AE and AE–trypsin complex.

The antioxidant activity of AE with and without trypsin were tested by DPPH and superoxide anion free radical scavenging assays. The results were shown in Fig. 9 and 10.

With the increase of trypsin concentration, the DPPH and superoxide anion scavenging rate of AE decreased gradually (*p* < 0.05), showing that trypsin had a significant effect on the antioxidant activity of AE. The antioxidant activity of AE with and without trypsin were tested by DPPH and superoxide anion free radical scavenging assays. The results were shown in Fig. 9 and 10. With the increase of trypsin concentration, the DPPH and superoxide anion scavenging rate of AE decreased gradually (*p* < 0.05), showing that trypsin had a significant effect on the antioxidant activity of AE. Franklin R. Vargas *et al.* Studied the inhibitory effect of emodin, AE and rhein on free radicals or reactive oxygen species in cell-free system by solid-state luminescence and luminol enhanced chemiluminescence and electronic absorption spectroscopy. It was found that emodin, AE and rhein had the following scavenging effects on the production of reactive oxygen species and free radicals: emodin > rhein > AE. This may be due to the different functional groups in the molecular structure of anthraquinones, which lead to the difference in the ability of electron capture and lead to the difference in the ability of scavenging free radicals.^38^ When anthraquinones containing different functional groups binded to the same protein, their binding sites and binding conformations were different. They also exhibited different biological activities.^39^ It was well known that phenolic hydroxyl groups were the main functional groups of phenols or substances containing phenolic hydroxyl groups exerted antioxidant capacity.^19^ We previously studied the interaction between emodin and pepsin in the same way, and found that the three phenolic hydroxyl groups in the molecule of aloe emodin formed hydrogen bonds with their surrounding amino acids respectively. Moreover, when pepsin was added into the reaction system of free radical scavenging, the ability of emodin in aloe emodin to scavenging free radicals decreased.^13^ In this paper, the interaction between emodin and trypsin was studied, and it was found that after the interaction between emodin and trypsin, two hydrogen bonds were formed between one phenolic hydroxyl group and its surrounding amino acid molecules. After adding trypsin into the antioxidant reaction system, the ability of aloe emodin to scavenging free radicals also decreased, which was similar to the results of previous studies. This may be due to the fact that the functional group-phenolic hydroxyl group in AE was affected by other groups, which led to the decrease of its ability of scavenging free radicals.

## 4. Conclusion

Overall, the findings outlined in the current study clearly describe the interaction of aloe emodin with trypsin. It was found that AE inhibited trypsin activity and decreased itself antioxidant activity. The interaction mechanism was studied by multi-spectral and molecular docking techniques. The results showed that the intrinsic fluorescence of trypsin could be quench by AE through static quenching mechanism, which confirmed the direct reaction between AE and trypsin. The reaction was spontaneous mainly through hydrogen bonding and van der Waals forces. In addition, AE binding brought about changes in α-helix, random coil, β-sheet, antiparallel β-sheet and β-turn structures of trypsin. This study also demonstrated the conformation of the AE–trypsin complex through molecular docking in order to further understand the interaction between trypsin and AE. Based on the current data, we conducted a comprehensive study on the interaction between trypsin and AE, which would be beneficial for the future application of AE in food industry.

## Funding

This work was supported by the National Natural Science Foundation of China (grant No. 31571800).

## Abbreviations

AE: Aloe-emodin
UV-vis: UV-visible
DPPH: 1,1-Diphenyl-2-picrylhydrazyl
Tris–HCl: Trisaminomethane–hydrochloric acid
Na_2_CO_3_: Sodium carbonate
FT-IR: Fourier transform infrared

## Conflicts of interest

The authors declare no competing financial interest.

## Supplementary Material
